# Survival outcomes in patients with human epidermal growth factor receptor 2 positive metastatic breast cancer administered a therapy following trastuzumab emtansine treatment

**DOI:** 10.1097/MD.0000000000022331

**Published:** 2020-09-18

**Authors:** Rurina Watanuki, Akihiko Shimomura, Shu Yazaki, Shoko Noda-Narita, Hitomi Sumiyoshi-Okuma, Tadaaki Nishikawa, Maki Tanioka, Kazuki Sudo, Tatsunori Shimoi, Emi Noguchi, Kan Yonemori, Kenji Tamura

**Affiliations:** a Department of Breast Surgery; b Department of Breast and Medical Oncology, National Cancer Center Hospital; c Department of Surgery, Keio University School of Medicine; d Department of Breast and Medical Oncology, National Global Health and Medicine; e Clinical Trial Management Section, Research Management Division, Clinical Research Support Office, National Cancer Center Hospital; f Department of Respiratory Medicine, The University of Tokyo Hospital, Tokyo, Japan.

**Keywords:** human epidermal growth factor receptor 2, metastatic breast cancer, progression-free survival, subsequent therapy, trastuzumab emtansine

## Abstract

Since 2013, trastuzumab emtansine (T-DM1) has been widely used in Japan to treat patients with human epidermal growth factor receptor 2 (HER2)-positive metastatic breast cancer (MBC) who were previously administered trastuzumab and a taxane. However, there is no information about the treatment outcomes after exposure to T-DM1 in Japanese patients with HER2-positive MBC. In this study, we aimed to describe the survival outcomes of patients with HER2-positive MBC who received a treatment following T-DM1 and clarify the predictive factors of their prognosis.

We retrospectively identified patients with HER2-positive MBC who received T-DM1 between April 1, 2014, and December 31, 2018, at the National Cancer Center Hospital, and focused on the population that received another line of therapy following T-DM1 discontinuation.

Thirty patients were available for the outcome analysis. Median progression-free survival (PFS) of the first subsequent therapy was 6.0 months [95% confidence interval (95% CI) 4.1–6.4], whereas the median overall survival (OS) from the first subsequent therapy was 20.6 months (95% CI 13.5 months to not reached). We divided the patients into 2 groups according to their PFS with T-DM1 treatment and compared their PFS with the subsequent therapy. The results revealed a significant difference in the median PFS with the first subsequent treatment between patients with the PFS of less than and more than 3 months [5.1 (95% CI 1.7–6.2) vs 6.2 (95% CI 4.0–11.3) months, *P* = .03].

This is the first study to evaluate the survival outcomes of post-T-DM1 therapy in Japanese patients with HER2-positive MBC. A short PFS with T-DM1 might affect the PFS with a treatment after T-DM1.

## Abstract 2

Supplemental Digital Content is available in the text

## 1 Introduction

Approximately 15% to 20% of breast cancers overexpress the human epidermal growth factor receptor 2 (HER2) protein.^[1]^ Trastuzumab (Tmab), a humanized monoclonal antibody directed against extracellular domain IV of HER2, is an example of a successful targeted therapy in breast cancer.^[2]^ Adding Tmab to the standard chemotherapy considerably improved the prognosis of patients with HER2-positive breast cancer.^[3]^ Despite this breakthrough, nearly all patients with HER2-positive metastatic breast cancer (MBC) eventually develop a progressive disease (PD) due to anti-HER2 therapy resistance.^[4]^ To overcome this clinical challenge, other HER2-targeted therapies, including lapatinib, pertuzumab (Pmab), and trastuzumab emtansine (T-DM1), have been developed.

Pmab is a monoclonal antibody that binds to HER2 on extracellular domain II, a different-domain trastuzumab.^[4]^ In the CLEOPATRA trial, superior progression-free survival (PFS) and overall survival (OS) was achieved by adding Pmab to Tmab and docetaxel therapy for patients with HER2-positive MBC who had not received anti-HER2 therapy or chemotherapy for metastatic disease.^[5,6]^ Therefore, this trial is considered a landmark trial that changed the field of clinical practice. Pmab was approved as a first-line treatment in combination with taxane and Tmab for HER-2 positive MBC.^[7]^

T-DM1 is an antibody–drug conjugate (ADC) composed of the cytotoxic agent, DM1, attached to Tmab via a stable thioether linker.^[8]^ In 2013, the United States and Japanese authorities approved T-DM1 for patients with HER2-positive MBC. In the EMILIA trial, there was a significant improvement in the PFS and OS of the T-DM1 arm compared with the capecitabine and lapatinib arm of patients who showed disease progression following one prior line of Tmab-containing therapy for HER2-positive MBC [median PFS, 9.6 vs 6.4 months, hazard ratio (HR) 0.65; median OS, 29.9 vs 25.9 months, HR 0.75].^[9,10]^ Another randomized phase III trial, the TH3RESA trial, compared T-DM1 with the physician's treatment choice in patients with HER2-positive MBC who were previously treated with Tmab and lapatinib. The median PFS was 6.2 months with T-DM1 and 3.3 months with the control [HR 0.53, 95% confidence interval (95% CI) 0.42–0.66; *P* < .0001].^[11]^ A trend favoring T-DM1 use was also recognized in the interim overall survival analysis (HR 0.552, 95% CI 0.369–0.826; *P* = .0034).^[11]^ Overall, improved survival outcomes have been reported in patients treated with T-DM1; however, there is a lack of information on treatment outcomes in patients with HER2-positive MBC after exposure to T-DM1. The current standard of care for HER2-positive MBC is a regimen consisting of Tmab and Pmab in the first-line setting, followed by T-DM1. Although both regimens have shown a survival benefit over the standard of care, there is no recognized standard of care after these therapies, and the subsequent therapies are poorly defined.

Recently, the phase III SOPHIA study of margetuximab in patients with HER2-positive MBC who were previously treated with ≥2 anti-HER2 therapies, including Pmab, revealed that margetuximab prolonged PFS compared with Tmab (5.8 vs 4.9 months, HR 0.76, 95% CI 0.59–0.98; *P* = .033) in the intent-to-treat analysis.^[12]^ Importantly, 90% of the patients in this study previously received T-DM1 treatment. Although the SOPHA trial showed the efficacy of margetuximab beyond the second-line treatment in patients with HER2-positive MBC, it did not include the Japanese population. Therefore, there is a lack of information regarding treatment outcomes in Japanese patients with HER2-positive MBC after exposure to T-DM1. In the present study, we aimed to describe the survival outcomes of patients with HER2-positive MBC administered a treatment following T-DM1 and clarify the predictive factors of their prognosis.

## 2 Patients and methods

### 2.1 Study population

We retrospectively identified patients with HER2-positive MBC who were administered T-DM1 between April 1, 2014, and December 31, 2018, at the National Cancer Center Hospital (NCCH). Patients categorized as HER2-positive had an immunohistochemistry score of 3+ and/or gene amplification ratio of ≥2 by fluorescence in situ hybridization. Patients who discontinued T-DM1 and received a therapy following T-DM1 treatment were enrolled in this retrospective study. Patients who were alive and continuing T-DM1 treatment, enrolled in clinical trials after T-DM1, and had occult primary cancer were excluded. The study was approved by the institutional review board of the NCCH. Because of the retrospective nature of the study, the requirement for informed consent was waived.

### 2.2 Data collection

Clinicopathological and survival data were obtained from the NCCH database. Medical records of each patient were reviewed to retrieve the following information: date of birth, the stage of initial breast cancer diagnosis (American Joint Committee on Cancer, seventh edition), performance status (PS), the site of metastases at the start of the first subsequent therapy, and tumor characteristics, including estrogen receptor (ER), progesterone receptor (PgR), and HER2 status of the primary tumor or metastatic tumor, if confirmed. ER, PgR, and HER2 were determined by the pathologist at our institution according to the 2010 and 2013 American Society of Clinical Oncology/College of American Pathologists Guidelines. The types of neoadjuvant and adjuvant therapies, including endocrine therapy, and prior chemotherapy regimens in the metastatic setting were recorded. The date when the first therapy following T-DM1 was started and ended and the date of death of patients (or last follow-up) were also recorded.

### 2.3 Outcomes and statistical analysis

Survival analyses were carried out according to PFS and OS. PFS was defined as the period from the first course of chemotherapy to disease progression or death by any cause. OS was defined as the period from the first course of chemotherapy to death from any cause or the last follow-up. The cutoff date for OS analysis was March 31, 2019. Best response was assessed according to Response Evaluation Criteria in Solid Tumors (RECIST) version.1.1. The Kaplan–Meier method was used to analyze survival rates. The univariate Cox proportional hazards regression model was used to analyze the association between the clinicopathological variables and PFS with the first therapy after T-DM1. All *P* values were 2-sided, and a *P* value of ≤.05 was considered to indicate significance. All statistical analyses were performed using EZR (Saitama Medical Center, Jichi Medical University), a graphical user interface for R (the R Foundation for Statistical Computing, version 3.4.1).^[13]^ More precisely, it is a modified version of R commander (version 2.4-1) that is designed to add frequently used statistical functions in biostatistics.^[13]^

## 3 Results

### 3.1 Patient characteristics

**Figure 1:**
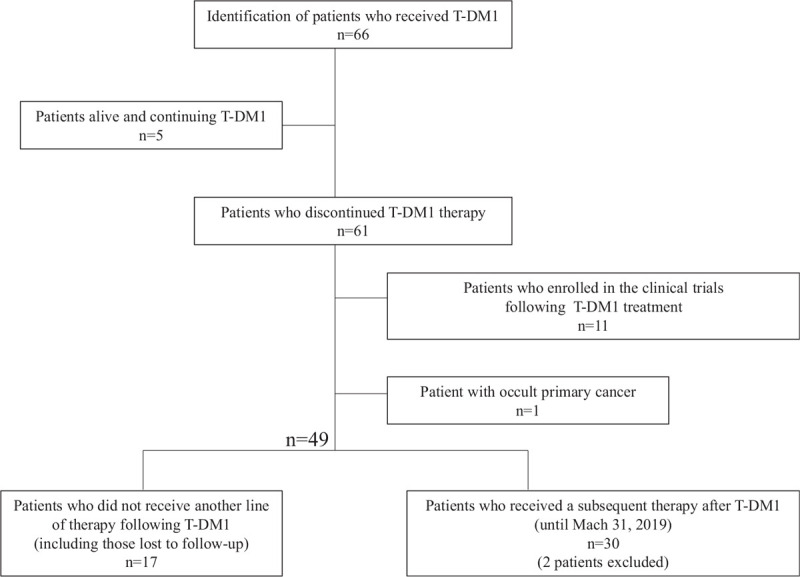
Flow chart of the study design. Sixty-six patients with HER-2 positive metastatic breast cancer were administered T-DM1 and 30 patients who received a therapy after T-DM1 were included in our analysis. HER2 = human epidermal growth factor 2, T-DM1 = trastuzumab emtansine.

Sixty-six patients with HER-2 positive MBC were administered T-DM1 between April 1, 2014, and December 31, 2018, at the NCCH. Sixty-one patients who discontinued the T-DM1 therapy, 11 patients who were enrolled in a clinical trial after T-DM1 treatment, and 1 patient with occult primary cancer were excluded from the study. Seventeen patients did not receive another line of therapy following T-DM1, including those lost to follow-up. Two patients were excluded because of incomplete data for analysis. Finally, 30 patients who received a therapy after T-DM1 discontinuation (ie, until March 31, 2019) were analyzed in this study (Fig. 1). The baseline characteristics of the study population at the start of the first therapy following T-DM1 treatment are presented in Table 1. The median age was 56 (30–80) years, and 29 patients (96.6%) had PS 0 to 1. At the initial diagnosis, 22 patients (73.3%) had stage I-III disease, and 8 patients (26.7%) had distant disease; 22 patients (73.3%) had ER and/or PgR-positive disease. Eighteen (60.0%) patients received neoadjuvant or adjuvant chemotherapy, 12 patients (40.0%) received adjuvant endocrine therapy, and 11 patients (36.7%) were exposed to Tmab in a neoadjuvant and/or adjuvant setting. Twenty-six patients (86.7%) had visceral disease, which was defined as a disorder of the lung, pleura, brain, liver, pancreas, duodenum, or adrenal gland. The median number of prior chemotherapy regimens for metastatic disease before the subsequent therapy was 2 (range 1–7). Furthermore, 13 (43.3%) patients received Pmab (Table 1). Eribulin monotherapy was the most common first subsequent therapy (33.3%) (Table 2). Thirteen (43.3%) patients received a regimen containing Tmab and/or lapatinib as the first therapy following T-DM1 treatment (Table 2). Ten patients (33.3%) did not receive another line of treatment after the first therapy following T-DM1.

**Table 1:**
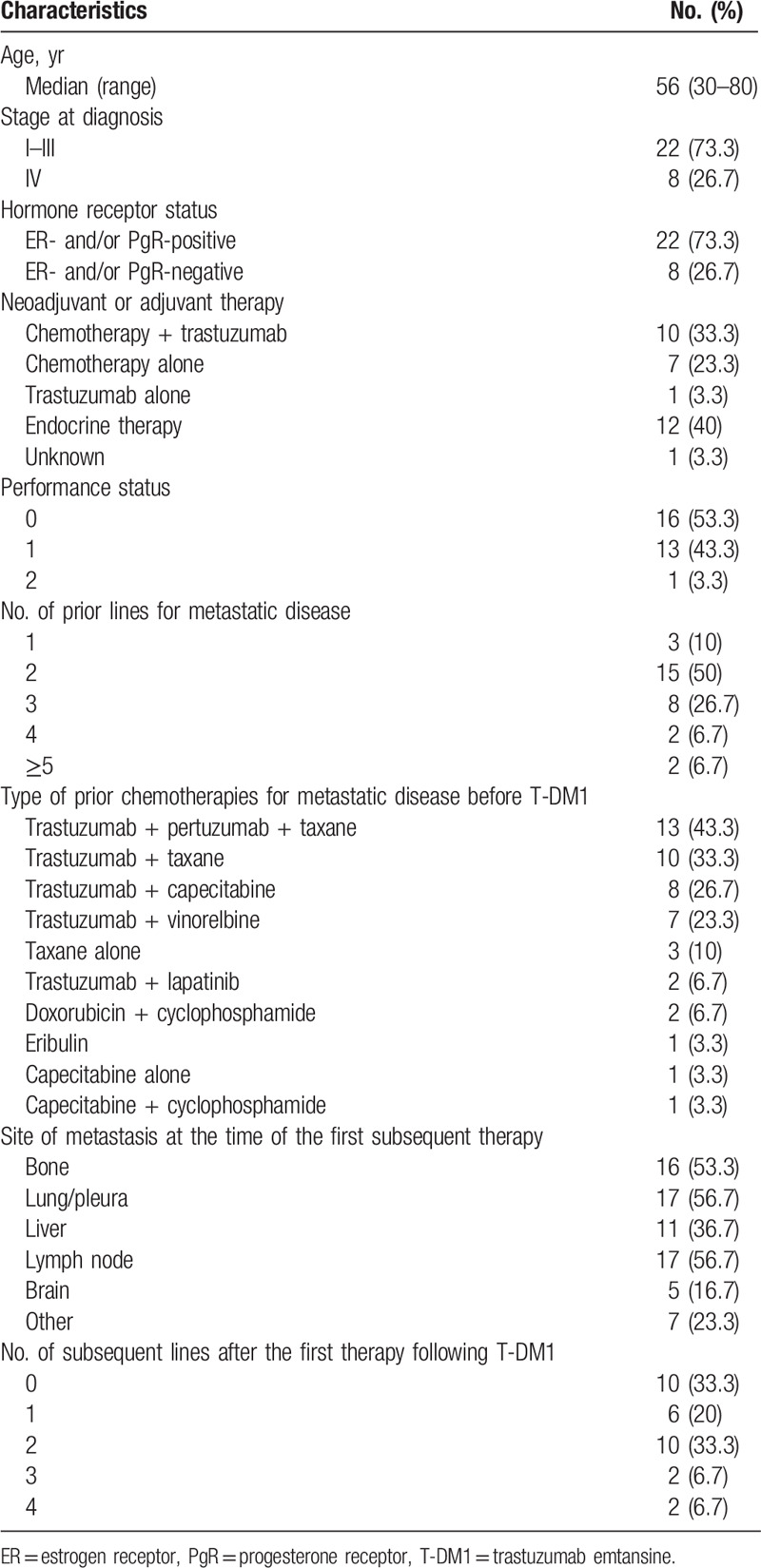
Characteristics of the study population (n = 30).

**Table 2:**
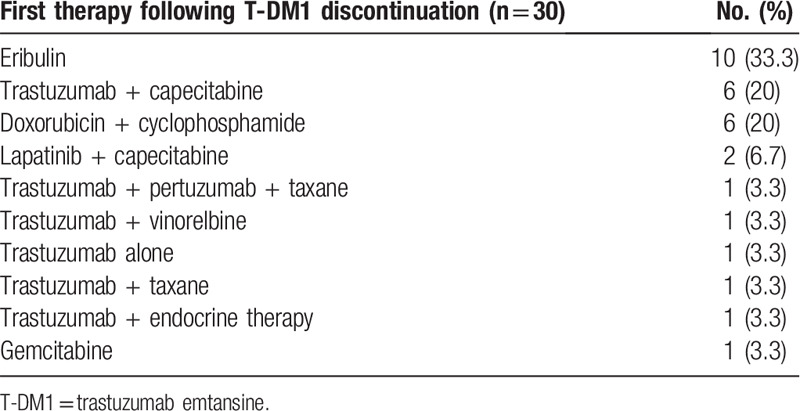
Chemotherapies administered after T-DM1 discontinuation.

### 3.2 Survival outcomes and efficacy

The median follow-up period was 21.8 months. The median PFS and median OS due to T-DM1 were 3.7 months (95% CI 2.7–5.5) and 28.9 months (95% CI 18.3 months to not reached), respectively (Supplementary Fig. 1a, http://links.lww.com/MD/E895 and 1b, http://links.lww.com/MD/E896). The best overall response to T-DM1 (n = 30) was as follows: 2 patients (6.7%) showed a partial response (PR), 16 (53.3%) showed a stable disease (SD), and 12 (40%) showed a PD.

**Figure 2:**
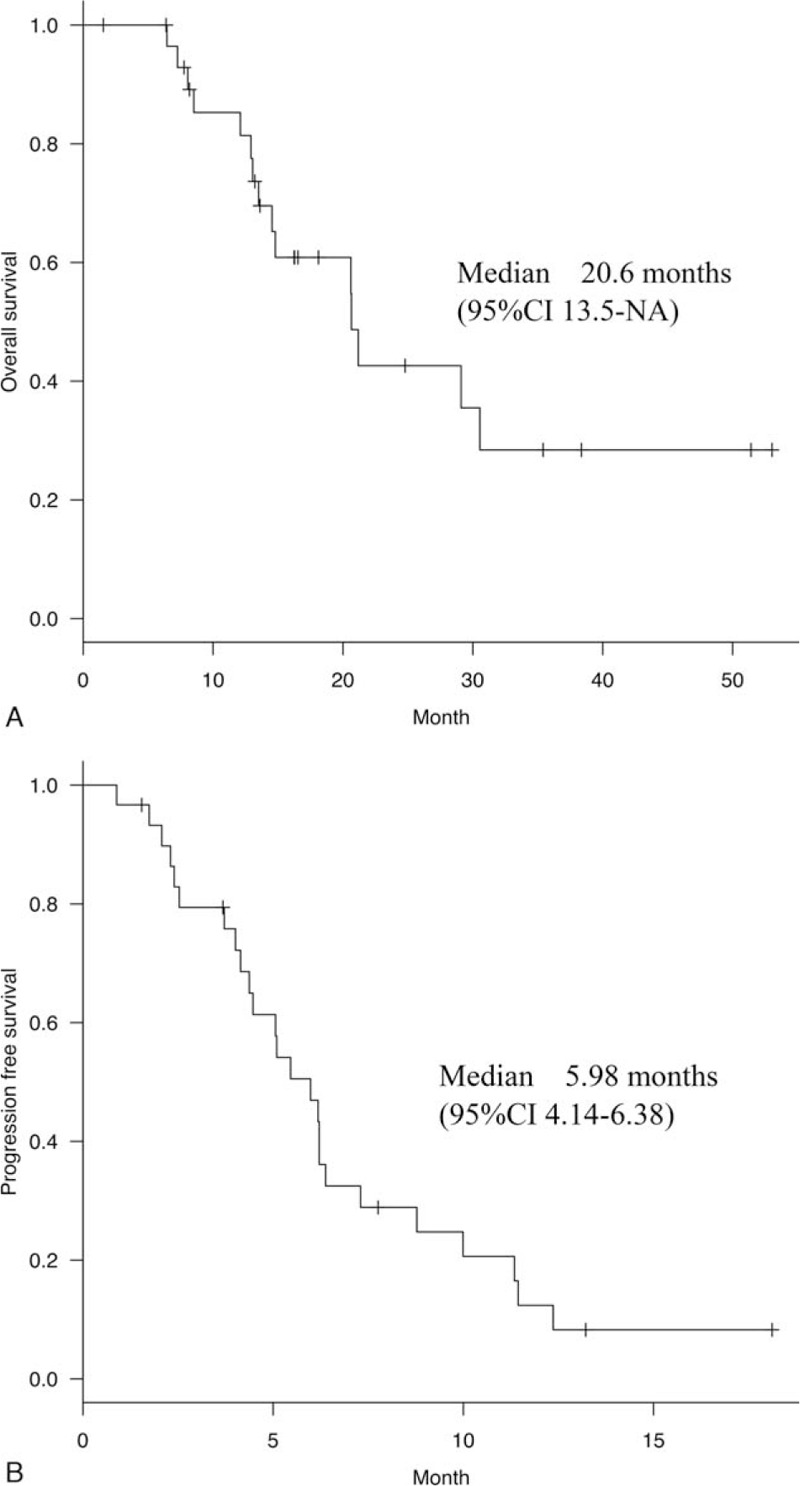
(A) Progression-free survival of patients with the first therapy after T-DM1. Median progression-free survival with the first subsequent therapy was 6.0 (95% CI 4.1–6.4) months. T-DM1 = trastuzumab emtansine. (B) Overall survival of patients with the first therapy after T-DM1. Median overall survival with the first subsequent therapy was 20.6 months (95% CI 13.5 months to not reached). T-DM1 = trastuzumab emtansine.

**Figure 3:**
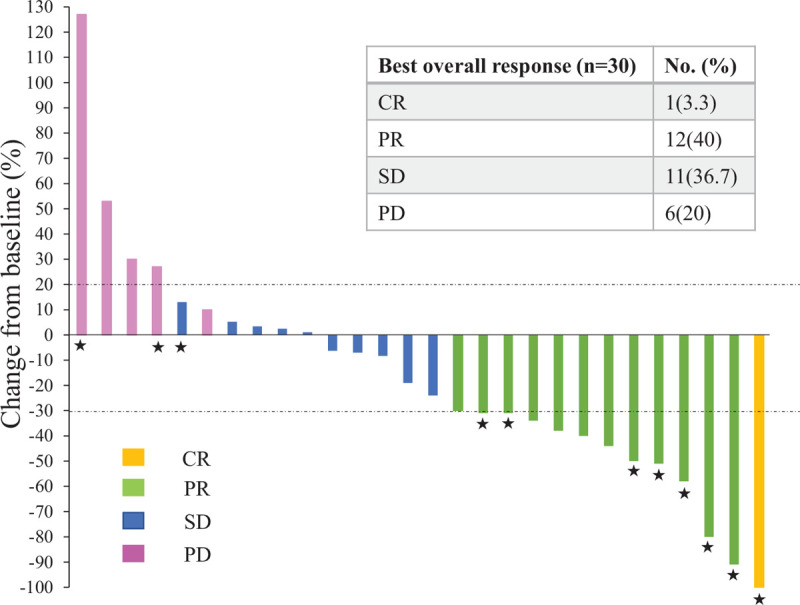
Waterfall plot of the best overall response for the target lesion of patients (Response Evaluation Criteria in Solid Tumors version 1.1). Yellow, green, blue, and pink indicate a complete response, partial response, stable disease, and progression disease, respectively. The upper and lower dotted lines represent a 20% increase and 30% reduction in tumor size, respectively. The asterisks indicate the patients who received anti-HER2 drugs in the first therapy after T-DM1. CR = complete response, PD = progression disease, PR = partial response, SD = stable disease.

The median PFS with the first subsequent therapy was 6.0 (95% CI 4.1–6.4) months (Fig. 2A), whereas the median OS from the initial administration of the first subsequent therapy was 20.6 (95% CI 13.5 months to not reached) (Fig. 2B). The best overall response to the first subsequent therapy (n = 30) was as follows: 1 patient (3.3%) showed a complete response, 12 (40%) showed a PR, 11 (36.7%) showed a SD, and 6 (20%) showed a PD. The objective responses of patients for the measurable target lesion are graphically presented in Figure 3 (n = 28). As shown in Figure 3, the response rates tended to be higher in the group that received anti-HER2 drugs as the first therapy after T-DM1 than in the group that did not receive (Supplemental Table 1, http://links.lww.com/MD/E897).

There was a significant difference in the medina PFS with T-DM1 between patients who received Pmab (n = 13) and those who did not (n = 17) [2.8 (95% CI 1.41–4.37) vs 4.8 (95% CI 2.73–7.82), *P* = .05]. In addition, the median PFS with the first subsequent treatment was shorter for patients administered Pmab before T-DM1 (n = 13) than those administered a regimen without Pmab (n = 17) [5.1 (95% CI 3.72–6.18) vs 6.2 (95% CI 2.53–11.4) months, *P* = .03]. We divided the patients into 2 groups according to their PFS with T-DM1 treatment and compared their PFS with the subsequent therapy. The results revealed a significant difference in the median PFS achieved with the first subsequent treatment between patients with a PFS of less than and more than 3 months [5.1 (95% CI 1.7–6.2) vs 6.2 (95% CI 4.0–11.3) months, *P* = .03].

The univariate analysis revealed that the prior use of Pmab (HR 2.70, 95% CI 1.09–6.66, *P* = .03), brain metastases (HR 5.28, 95% CI 1.69–16.5, *P* < .004), and the PFS with T-DM1 (HR 2.64, 95% CI 1.08–6.47, *P* = .03) were significant predictive factors of the PFS of patients with HER2-positive MBC administered the first subsequent therapy (Table 3). The hormone receptor status, number of prior lines, and use of anti-HER2 drugs as the first post-T-DM1 therapy were not associated with the PFS attained with the first subsequent therapy.

**Table 3:**
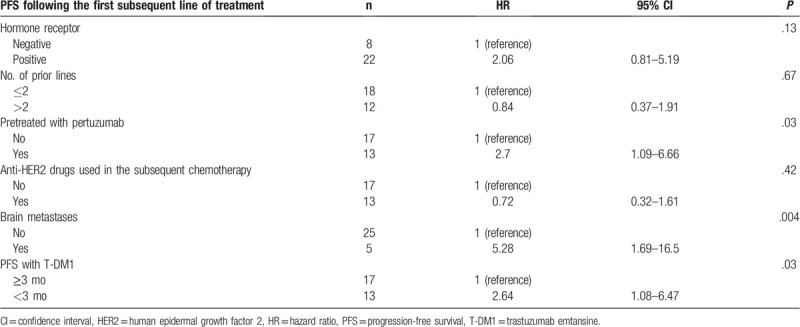
Results of the univariate analysis.

## 4 Discussion

To the best of our knowledge, this is the first study to report the survival outcomes of Japanese patients with HER2-positive MBC who received a therapy following T-DM1 administration. The median PFS with the first subsequent therapy was prolonged to 6.0 months following the discontinuation of T-DM1 in patients with HER2-positive MBC. This result is similar to that reported by Olson et al,^[14]^ who found that the median duration to the first subsequent regimen was 5.5 months in 15 patients with HER2-positive MBC who received another line of therapy following T-DM1. Here, we found that the median OS of heavily treated patients was 20.6 months with the first subsequent therapy. Thus, our survival data support the improved OS of all patients with MBC over the past 10 to 15 years, and it might be related to the development of anti-HER2 target therapy and supportive care.^[15,16]^

In the present study, patients who received Pmab before T-DM1 had a significantly poor PFS with both T-DM1 and first subsequent therapy than those administered a regimen without Pmab. Previously, we reported that Japanese patients with HER2-positive MBC and treated with Tmab and Pmab before T-DM1 received fewer benefits from T-DM1. The results of our retrospective study demonstrated that the median PFS was significantly shorter in the Tmab/Pmab group (patients administered Tmab and Pmab before T-DM1) than the Tmab group (patients administered only Tmab before T-DM1) (2.8 vs 4.8 months) and the best response was lower in the Tmab/Pmab group (11.1% vs 25.0%).^[17]^ The results of other retrospective studies are consistent with our previous data. Dzimitrowicz et al^[18]^ reported a low tumor response rate and a short median duration of T-DM1 therapy in patients previously treated with Pmab and Tmab; these findings are less favorable than the objective tumor response rates and PFS reported in clinical trials with T-DM1. They suggested a clinically relevant benefit in patients with HER2-positive MBC who experienced progression when prior Pmab- and Tmab- containing therapies were administered. Similar findings were reported by Vici et al,^[19]^ who demonstrated the efficacy of T-DM1 in patients with HER2-positive MBC treated in a real-world practice, and its activity in Pmab-pretreated patients. In patients pretreated with Pmab and administered T-DM1 as a second-line therapy, the median PFS was 3 months. Conversely, patients who did not receive previous therapy and administered other anti-HER2 target therapies and T-DM1 as a second-line therapy had the median PFS of 8 months (*P* = .0001).^[19]^ The results of this study are similar to those of previous studies, suggesting that not only the efficacy of T-DM1 after Pmab but also the clinical outcomes of the treatment administered after T-DM1 might be affected by prior Pmab therapy.

Here, we found that the PFS of patients treated with T-DM1 is one of the significant predictive factors of the PFS with the first subsequent therapy. In fact, patients with HER2-positive MBC with a PFS of more than 3 months with T-DM1 treatment had better survival outcomes with the subsequent therapy than those with a PFS of less than 3 months with T-DM1 treatment. As the median PFS of patients treated with T-DM1 was 3 months in this study, it was set as the cutoff value. Generally, patient response to a treatment is evaluated every 3 months in our clinical practice, and those who experience disease progression in the first 3 months from the initiation of T-DM1 treatment might have a worse prognosis. We considered the possibility that a short PFS of 3 months with T-DM1 may be affected by prior Pmab administration, as mentioned above.

In the present study, patients treated with T-DM1 presented a short PFS, a finding that is inconsistent with that of previous studies. The difference could be because of the following: the effect of pre-treatment with Pmab before T-DM1 and resistance to T-DM1. Although the resistant mechanism to T-DM1 has been discussed, it has not been completely understood. The expression of HER2 in cancer cells is essential for the efficacy of T-DM1, but tumor heterogeneity may prevent a response. Poor HER2-DM1 complex internalization, defective intracellular and endosomal trafficking of the HER2-DM1 complex, defective lysosomal degradation of T-DM1, and drug efflux proteins, such as MDR1, are among the mechanisms of T-DM1 resistance.^[20,21]^ Following the approval of T-DM1, ADC development has become a crucial therapeutic strategy for HER2-positive breast cancer. When a receptor is more specific to a cancer cell relative to a normal cell, ADC is less toxic and more effective. Thus, to overcome the resistant mechanisms of T-DM1, developing new ADCs for HER2 target therapy when T-DM1 treatment leads to disease progression is of great interest to researchers.

Using an original linker-payload technology, a new HER2-targeting ADC composed of the humanized anti-HER2 antibody trastuzumab deruxtecan (DS-8201a) was designed and attached via a cleavable peptide-based linker to a potent topoisomerase I inhibitor payload.^[22]^ DS-8201a has the same amino acid sequence as Tmab,^[22]^ and in a dose-escalation phase I study, it was demonstrated to exhibit antitumor activity and a favorable toxicity profile in patients with advanced solid tumors, including breast and gastric cancers, and with low-HER2-expressing tumors.^[23]^ In a subsequent dose-escalation and dose-expansion phase I trial in patients with HER2-positive MBC and previously treated with T-DM1, DS8201a displayed promising activity with manageable safety in patients with a median of seven previous cancer therapies, 60% of which helped achieve an objective response.^[24]^ These studies indicated that various factors might contribute to the increased activity of DS8201a compared with other anti-HER2 therapies, such as T-DM1.^[24]^ DS8201a has a drug-to-antibody ratio (DAR) of 7 to 8, facilitating a higher delivery of payload to target cells, whereas the DAR of T-DM1 is 3.5.^[22]^ In addition, DS8201a exhibits a bystander killing effect in heterogeneous tumor models, in which both HER2-positive cells and neighboring HER2-negative cells incur toxicity,^[25]^ and this might be effective even in patients with low HER2-expression tumors or tumors that are resistant to anti-HER2 therapies.

This study had some limitations. First, because most patients with HER2-positive MBC who discontinued T-DM1 therapy were participants in the clinical trials, the sample size of the present retrospective study was small. Second, in the univariate analysis, brain metastasis was a significant factor related to the PFS of patients who received a therapy after T-DM1. However, we cannot imply that it reflects the therapeutic effect of the subsequent treatment. Compared with other intrinsic subtypes, HER2-positive breast cancer has a high propensity to metastasize to the brain.^[26]^ In fact, it is estimated that 35% to 55% of patients with HER2-positive breast cancer will develop brain metastases during the course of the disease, and they are known to have a poor prognosis.^[27,28]^

To the best of our knowledge, this is the first study to identify the PFS and OS of Japanese patients with HER2-positive MBC who were administered a therapy following T-DM1 treatment. In addition, brain metastases, the prior use of Pmab, and the PFS with T-DM1 treatment were demonstrated to be significantly associated with the PFS of patients with HER2-positive MBC administered a treatment following T-DM1. Owing to the development of specific targeted therapies, particularly the introduction of a double blockage using Tmab and Pmab, followed by T-DM1 in the first-line setting, the survival outcomes of these patients have substantially improved.^[29]^ Despite such important therapeutic advances, most patients will relapse and exhibit resistance to their therapies.^[29]^ Although the data obtained in the present study was from are relatively small sample, and a randomized design was not employed, we believe that the present real-world data will contribute to the development of treatment strategies for HER2-positive MBC. We anticipate that the resistant mechanisms of anti-HER2 targeted therapy will be clarified and a new treatment option will be established for patients with HER-2-positive MBC pretreated with Pmab and T-DM1 as a salvage-line treatment.

## Acknowledgments

We would like to thank Dr. Yukari Uemura, Center for Clinical Sciences, National Center for Global Health and Medicine, for her helpful and expertise on the statistical methods.

## Author contributions

R.W., A.S. and S.Y. designed the study. R.W. reviewed clinical information. R.W. and A.S. performed the statistical analyses. R.W. and A.S. wrote the manuscript. A.S., T.S. critically revised the manuscript. All authors read and approved the manuscript.

## Abbreviations

Abbreviations: ADC = antibody–drug conjugate, CI = confidence interval, DAR = drug-to-antibody ratio, DS-8201a = trastuzumab deruxtecan, ER = estrogen receptor, HER2 = human epidermal growth factor receptor 2, HR = hazard ratio, MBC = metastatic breast cancer, NCCH = National Cancer Center Hospital, OS = overall survival, PD = progressive disease, PFS = progression-free survival, PgR = progesterone receptor, Pmab = pertuzumab, PR = partial response, PS = performance status, RECIST = Response Evaluation Criteria in Solid Tumors, SD = stable disease, T-DM1 = trastuzumab emtansine, Tmab = trastuzumab.
